# Removal of a Maxillary Tumor by Means of the Dental Engine

**Published:** 1883-08

**Authors:** 


### REMOVAL OF A MAXILLARY TUMOR BY MEANS OF THE DENTAL ENGINE.

Mr. F. A. Nixon successfully removed an osseous tumor of the upper jaw by means of this instrument last Saturday, in Mercer's Hospital. The patient was a young country girl, and the tumor, which caused considerable deformity, extended upward to the floor of the orbit, and backward to the pterygo-maxillary fosse. A great and important advantage in the operation, as performed by Mr. Nixon, was that the small circular steel saws used were employed from the mouth, no incision having been made in the cheek, and an unsightly cicatrix consequently avoided. The operation occupied one hour and ten minutes in performance. No difficulty was met with in using the saws, which, being so small in diameter, one quarter and one half inch respectively, were readily worked in a limited space, and could be guided by touch alone. This, in a difficult accessible region such as the pterygo-maxillary fosse, is an advantage of no little importance. The patient is progressing favorably.—*British Med. Journal.*

